# Estimating the number of symptomatic SARS-CoV-2 infections among vaccinated individuals in the United States—January–July, 2021

**DOI:** 10.1371/journal.pone.0264179

**Published:** 2022-03-09

**Authors:** Kiersten J. Kugeler, John Williamson, Aaron T. Curns, Jessica M. Healy, Leisha D. Nolen, Thomas A. Clark, Stacey W. Martin, Marc Fischer

**Affiliations:** Centers for Disease Control and Prevention (CDC), COVID-19 Response Team, Fort Collins, Colorado, United States of America; University "Magna Graecia" of Catanzaro, ITALY

## Abstract

As of March 2021, three COVID-19 vaccines had been authorized by the U.S. Food and Drug Administration (FDA) for use in the United States. Each has substantial efficacy in preventing COVID-19. However, as efficacy from trials was <100% for all three vaccines, disease in vaccinated people is expected to occur. We created a spreadsheet-based tool to estimate the number of symptomatic COVID-19 cases among vaccinated people (vaccine breakthrough infections) based on published vaccine efficacy (VE) data, percent of the population that has been fully vaccinated, and average number of COVID-19 cases reported per day. We estimate that approximately 199,000 symptomatic vaccine breakthrough infections (95% CI: ~183,000–214,000 cases) occurred in the United States during January–July 2021 among >156 million fully vaccinated people. With high SARS-CoV-2 transmission and increasing numbers of people vaccinated in the United States, vaccine breakthrough infections will continue to accumulate. Understanding expectations regarding number of vaccine breakthrough infections enables accurate public health messaging to help ensure that the occurrence of such cases does not negatively affect vaccine perceptions, confidence, and uptake.

## Introduction

Widespread uptake of safe and effective vaccines is critical to controlling the COVID-19 pandemic. Three COVID-19 vaccines have been authorized or approved by the U.S. Food and Drug Administration (FDA) for use in the United States, including the 2-dose Pfizer-BioNTech BNT162b2 mRNA and Moderna mRNA-1273 vaccines and the single-dose adenovirus-based Johnson & Johnson/Janssen Ad.26.COV2.S vaccine [1–3]. In large, randomized controlled trials, the Pfizer-BioNTech and Moderna mRNA vaccines each had an efficacy of ≥94% in preventing symptomatic, laboratory-confirmed COVID-19 following the 2-dose series [4, 5]. Among the over 32,000 people who received either the Pfizer-BioNTech or Moderna vaccine during those clinical trials, 20 developed COVID-19 after vaccination. Among the 19,514 people randomized to receive Janssen vaccine during those trials, 116 developed COVID-19 following vaccination, resulting in an efficacy of 67% against moderate-to-severe COVID-19 [6]. The Pfizer-BioNTech and Moderna vaccines were authorized by FDA and recommended by the Advisory Committee of Immunization Practices for use in the United States in December 2020 [2, 3, 7, 8], while the Janssen vaccine was authorized and recommended for use at the end of February 2021 [1, 9]. Vaccine administration in the United States began within a few days of authorization for each vaccine.

As no vaccine is 100% effective at preventing illness, COVID-19 occurring among vaccinated people, often referred to as vaccine breakthrough infections, are expected. Amid increases in vaccination coverage in the setting of widespread SARS-CoV-2 transmission, the numbers of COVID-19 cases among vaccinated people could be substantial. We estimated the number of symptomatic vaccine breakthrough infections expected in the United States based on published vaccine efficacy (VE) data, percent of the population that had been fully vaccinated, and reported COVID-19 case counts.

## Methods

We developed a tool in Microsoft Excel^©^ to estimate the expected number of symptomatic COVID-19 cases among vaccinated persons per day in the United States using publicly available data. Inputs are the 7-day moving average for daily numbers of COVID-19 cases in the United States as reported to the Centers for Disease Control and Prevention (CDC), the cumulative number of persons fully vaccinated with each vaccine as reported to CDC as of 14 days prior to each 7-day average case count, and VE data from phase 3 trials of the three vaccines authorized in the United States [1–3, 10, 11]. The number of symptomatic vaccine breakthrough infections, rather than all symptomatic and asymptomatic vaccine breakthrough infections, were calculated because prevention of symptomatic disease was the primary phase 3 clinical trial endpoint reported for the COVID-19 vaccines authorized in the United States and corresponds to published vaccine efficacy figures. As this project incorporates secondary use of publicly available data, human subjects research review was deemed unnecessary.

Breakthrough cases were defined as those occurring in persons ≥14 days after completion of vaccination with an authorized COVID-19 vaccine, a delay to reflect when maximum immunity conveyed by vaccination is reached. Given the similar reported VE from clinical trials for the Pfizer-BioNTech and Moderna vaccines, the average VE of 94.6% was used for both mRNA vaccines in the calculator [4, 5], while 66.9% VE was used for the Janssen vaccine [1]. Calculations were restricted to persons aged ≥18 years for January through May, the primary population that received vaccines during that time; the population reflected in calculations was expanded to approximate persons aged ≥12 years beginning at the end of May. Available data suggest that 87.4% of reported U.S. cases to date as of the end of July had occurred among persons aged ≥18 years, and 93.7% of total cases had occurred among persons aged ≥12 years [12]. We approximated the average number of COVID-19 cases occurring per day among the vaccine eligible population accordingly. We also proportionally restricted the denominator data used to approximate the proportion of the vaccine eligible population that was fully vaccinated per 2019 U.S. census estimates [12]. The number of persons fully vaccinated with Janssen vaccine registered as of 14 days prior to each date for case count ascertainment was subtracted from the total number of persons vaccinated as of that date to approximate number of persons vaccinated with Pfizer-BioNTech or Moderna vaccines at that time [11].

The number of symptomatic vaccine breakthrough infections expected per day is a function of VE and vaccination coverage in the population. For these calculations, *C* denotes the approximated 7-day moving average for daily number of reported COVID-19 cases among the vaccine eligible population and *V* represents different vaccination “groups” according to numeric subscripts: 0 for unvaccinated or not fully vaccinated, 1 for Janssen vaccine, and 2 for Moderna and Pfizer-BioNTech vaccines; *%V* is the percent of the population fully vaccinated in each vaccine group. VE is calculated as (1 –the risk ratio [RR]), where RR is the ratio of confirmed symptomatic SARS-CoV-2 infections per 1000 person-years among those receiving vaccine in phase 3 trials divided by those receiving placebo. The Janssen RR_1_ was 0.331 (VE_1_ = 0.669), the Pfizer-BioNTech and Moderna RR_2_ was 0.054 (VE_2_ = 0.946), while RR_0_ was defined as 1 (VE_0_ = 0) for people who are unvaccinated or not fully vaccinated. The expected number of symptomatic vaccine breakthrough infections per day is calculated as:

$$\frac{C\left(\hat{{RR}_{1}}*\%V_{1}\right)+\;\left(\hat{{RR}_{2}}*\%V_{2}\right)}{{\%V}_{0}+\;\left(\hat{{RR}_{1}}*\%V_{1}\right)+\;\left(\hat{{RR}_{2}}*\%V_{2}\right)}$$

Variance was calculated based on available phase 3 clinical trial data for the Pfizer-BioNTech and Moderna vaccine trials using Poisson regression models (S1 File). The pooled variance of the expected symptomatic vaccine breakthrough infections was estimated to be $\text{Var}({\hat{\beta }}_{1})=0.01149$, $\text{Var}({\hat{\beta }}_{2})=0.05551$, with $\text{Cov}({\hat{\beta }}_{1},{\hat{\beta }}_{2})=0$ and calculated as

$$C^{2}\frac{\left(\hat{RR_{1}}*\text{\%}V_{0}*\text{\%}V_{1}\right)}{{\left\{\left[\text{\%}V_{0}+\;\left(\hat{RR_{1}}*\text{\%}V_{1}\right)+\;\left(\hat{RR_{2}}*\text{\%}V_{2}\right)\right]*2\right\}}^{2}}\;\text{Var}{\hat{\beta }}_{1}+\;C^{2}\frac{\left(\hat{RR_{2}}*\text{\%}V_{0}*\text{\%}V_{2}\right)}{{\left\{\left[\text{\%}V_{0}+\;\left(\hat{RR_{1}}*\text{\%}V_{1}\right)+\;\left(\hat{RR_{2}}*\text{\%}V_{2}\right)\right]*2\right\}}^{2}}\;\text{Var}{\hat{\beta }}_{2}$$

The first persons in the United States to be vaccinated against SARS-CoV-2 completed their 2-dose series during the week of January 4, 2021. Therefore, we began calculating the expected number of symptomatic vaccine breakthrough infections 14 days later, the week beginning January 17. We calculated weekly estimates using approximated average case counts among the vaccine eligible population and vaccination coverage as of the Sunday beginning each week and then multiplied the daily estimate by seven. We estimated per-week symptomatic breakthrough infections through the last week of July. Incorporation of Janssen vaccine into the estimates began in mid-March. Case counts and eligible population included persons aged 12yearss during the week beginning May 30. We calculated cumulative expected counts to date by summing weekly expected vaccine breakthrough case counts. We derived 95% confidence intervals (CI) around cumulative counts by summing weekly variances as described above into standard CI calculations.

To understand the relative influence of community transmission and VE in determining the number of expected vaccine breakthrough infections, we calculated expected cumulative counts during the same time period under two hypothetical scenarios: 1) doubling the average daily COVID-19 case counts each week; and 2) modifying population vaccination coverage during January–July such that it entirely reflected VE of 67%, VE associated with the Janssen vaccine.

## Results

**Fig 1:**
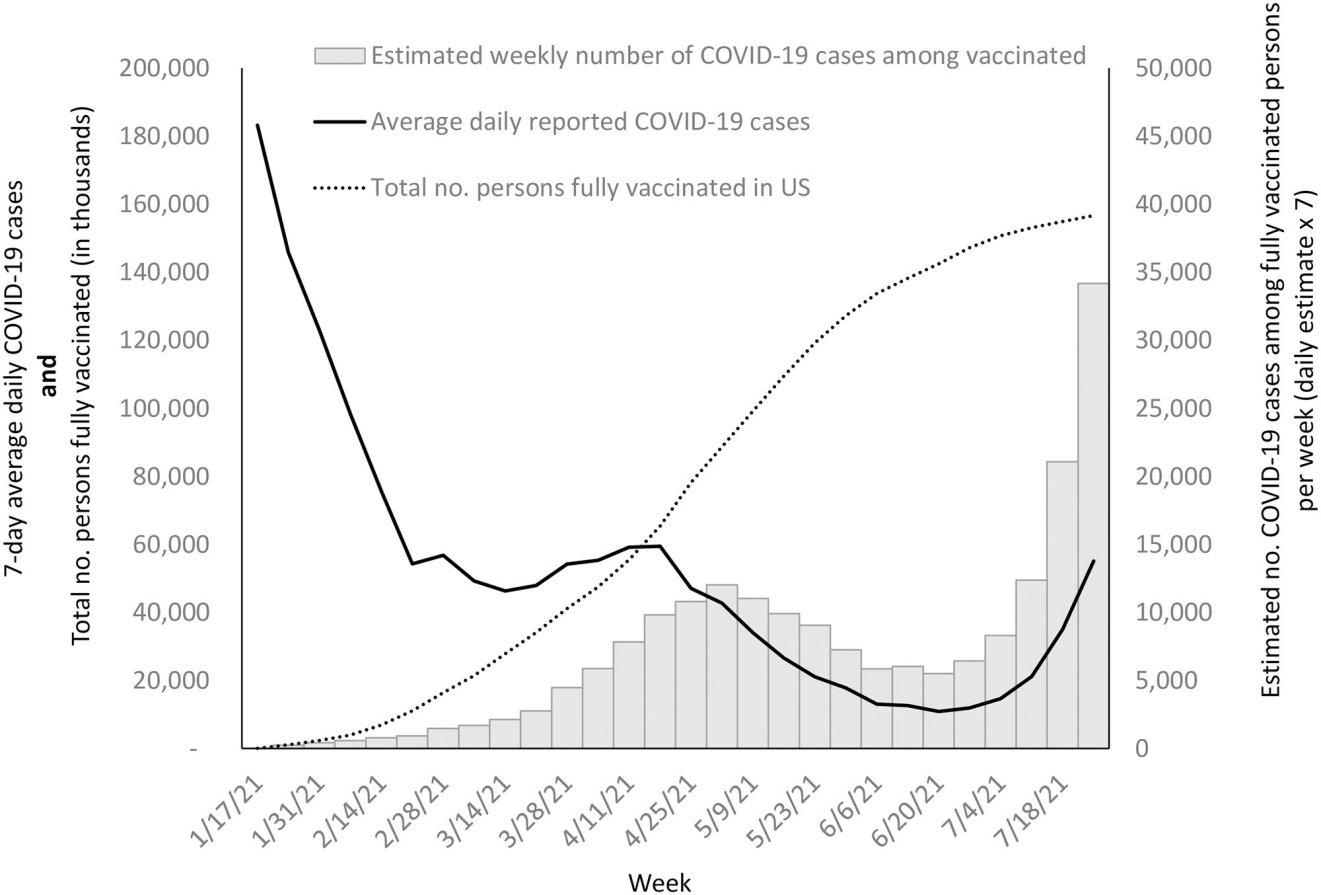
Estimated number of symptomatic COVID-19 cases occurring among the vaccinated population, average daily COVID-19 cases among vaccine eligible age groups, and number of persons fully vaccinated per week in the United States, January–July 2021.

Nearly 12 million COVID-19 cases were reported in the United States during January–July 2021 [10]. The number of COVID-19 cases reported per day during this period ranged from a high of approximately 210,000 cases in mid-January to a low of approximately 12,000 cases in late June [10]. The estimated number of symptomatic vaccine breakthrough infections in the United States ranged from a low of almost two per day (11 per week) in January to nearly 5,000 per day (34,000 per week) during the last week of July (Table 1 and Fig 1).

**Table 1:** COVID-19 case counts, vaccine coverage, and estimated number of expected symptomatic vaccine breakthrough infections, by week—United States, January–July 2021.

Week start date	Average daily COVID-19 case counts*	Full vaccination coverage as of 2 weeks prior**	Estimated number of symptomatic vaccine breakthrough infections	
			Per day (95% CI)	Cumulative (95% CI)
Jan 17	183,231	0.02%	2 (1–2)	–
Jan 24	145,842	0.44%	35 (19–51)	253 (141–365)
Jan 31	122,914	0.91%	61 (33–89)	682 (454–909)
Feb 7	98,367	1.55%	84 (45–122)	1,266 (914–1,619)
Feb 14	75,756	2.70%	113 (61–165)	2,059 (1,551–2,567)
Feb 21	54,255	4.35%	133 (72–194)	2,989 (2,324–3,653)
Feb 28	56,771	6.43%	210 (113–307)	4,458 (3,510–5,407)
Mar 7	49,249	8.40%	243 (131–354)	6,158 (4,930–7,386)
Mar 14	46,334	10.92%	305 (165–445)	8,291 (6,721–9,862)
Mar 21	47,885	13.35%^†^	395 (214–3576)	11,058 (9,040–13,075)
Mar 28	54,183	16.13%^†^	638 (391–885)	15,524 (12,866–18,183)
Apr 4	55,346	18.60%^†^	839 (545–1,113)	21,395 (18,032–24,757)
Apr 11	59,161	21.75%^†^	1,118 (740–1,496)	29,222 (24,944–33,499)
Apr 18	59,415	25.62%^†^	1,402 (937–1,867)	39,038 (33,663–44,413)
Apr 25	47,047	30.64%^†^	1,542 (1,084–2,001)	49,833 (43,572–56,094)
May 2	42,752	34.74%^†^	1,718 (1,224–2,211)	61,857 (54,706–69,009)
May 9	34,010	38.87%^†^	1,575 (1,111–2,040)	72,886 (65,031–80,741)
May 16	26,571	42.91%^†^	1,416 (992–1,839)	82,795 (74,399–91,190)
May 23	21,060	46.71%^†^	1,292 (908–1,677)	91,841 (83,024–100,657)
May 30	17,804	45.40%^†^	1,037 (726–1,347)	99,097 (90,016–109,178)
June 6	13,036	47.74%^†^	836 (590–1,083)	104,950 (95,707–114,193)
June 13	12,603	49.32%^†^	861 (609–1,112)	110,976 (101,566–120,385)
June 20	10,883	50.84%^†^	786 (557–1,015)	116,479 (106,934–126,024)
June 27	11,925	52.54%^†^	919 (653–1,185)	122,911 (113,186–132,636)
July 4	14,640	53.78%^†^	1,187 (847–1,526)	131,218 (121,207–141,229)
July 11	21,117	54.63%^†^	1,767 (1,263–2,271)	143,587 (132,971–154,202)
July 18	34,955	55.28%^†^	3,012 (2,160–3,863)	164,668 (152,493–176,843)
July 25	55,105	55.90%^†^	4,882 (3,513–6,251)	198,840 (183,346–214,333)

CI = Confidence intervals;

*Average daily COVID-19 cases among vaccine eligible population, defined as ≥18 years of age through May and ≥12 years of age from end of May on. As exact proportions per age group over time are unavailable, these were approximated as 87.4% of total and 93.7% of total, as is the proportional makeup over the duration of the pandemic.

**Proportion of the eligible population fully vaccinated (completion of an FDA-authorized vaccine or vaccine series) as of 14 days prior to date noted

^†^includes 0.5%-4.6% of eligible population vaccinated with the Janssen vaccine.

As of the end of July, we estimate that a total of 198,840 symptomatic vaccine breakthrough infections (95% CI: 183,346–214,333 cases) occurred in the United States among >156 million fully vaccinated people (Table 1 and Fig 1). On average, starting in February, the number of expected vaccine breakthrough infections increased by 37% each week, but slowed beginning the last week of April amid falling numbers of COVID-19 cases in the United States. This trajectory in the number of expected symptomatic vaccine breakthrough cases each week shifted rapidly with the increasing SARS-CoV-2 transmission driven by the spread of the Delta variant beginning in late June (Fig 1) [10]. The number of expected vaccine breakthrough infections during that time translates to a cumulative incidence of approximately 127 vaccine breakthrough infections per 100,000 fully vaccinated people.

The expected number of vaccine breakthrough infections varied substantially under different hypothetical scenarios reflective of 1) doubling daily average case counts, and 2) all vaccination occurring at VE of 67% (Table 2). The relationship between COVID-19 cases and the expected number of vaccine breakthrough infections was proportional (i.e., when case counts doubled, so did symptomatic vaccine breakthrough infections), whereas VE had far more influence on the expected number of symptomatic vaccine breakthrough infections. Compared to vaccination with an average VE of nearly 95%, as occurred during January through July in the United States, a hypothetical scenario in which all vaccination occurred at VE of about 67% nearly quadrupled the number of expected symptomatic vaccine breakthrough infections without modifying other parameters.

**Table 2:** Expected cumulative number of symptomatic COVID-19 vaccine breakthrough infections under differing hypothetical scenarios of disease incidence, vaccination coverage, and vaccine efficacy—United States, January–July 2021.

Scenario	Estimated cumulative number of symptomatic breakthrough infections (95% CI)	Percent change from baseline
Baseline*	198,840 (183,346–214,333)	–
Scenario 1: Case counts doubled each week from baseline	397,679 (366,693–428,666)	100%
Scenario 2: Population vaccination coverage entirely with a vaccine with 67% VE	759,019 (728,218–789,820)	382%

CI = Confidence intervals; VE = Vaccine efficacy

*Estimated cumulative number of expected symptomatic vaccine breakthrough cases as of the week beginning July 25, 2021.

## Discussion

We created a spreadsheet-based calculator to estimate the number of symptomatic vaccine breakthrough infections in the United States based on the average number of COVID-19 cases, percent of the population fully vaccinated, and published efficacies of the three FDA-authorized vaccines. Using this tool, we estimate that approximately 200,000 symptomatic SARS-CoV-2 vaccine breakthrough infections occurred by the end of July among the over 156 million people fully vaccinated in the United States by the middle of July. Vaccine breakthrough infections occur in only a small fraction of all vaccinated persons. Understanding the number of expected vaccine breakthrough infections is important for accurate public health messaging to help ensure that the occurrence of such cases does not negatively affect vaccine perceptions, confidence, and uptake.

We developed this tool incorporating ideal VE scenarios. Real-world vaccine effectiveness may be lower, particularly for people who are older or have underlying health conditions [13, 14]. Lower effectiveness also may result from decreased protection against certain SARS-CoV-2 variants, including the Delta variant. Early vaccine effectiveness studies in the United States and elsewhere demonstrated high effectiveness of mRNA vaccines against symptomatic infection and severe disease in various real-world situations [15, 16], including approximately 95% effectiveness among large cohorts of healthcare workers and >85% among residents of skilled nursing facilities [17–22]. However, more recent estimates of vaccine effectiveness against infection with the Delta variant have decreased, while effectiveness remains high against hospitalization and death [23, 24]. Although we used efficacy data from clinical trials as inputs to estimate expected vaccine breakthrough infections, these inputs could be updated using additional vaccine effectiveness data as those become increasingly available.

COVID-19 vaccines have demonstrated the ability to mitigate risk of severe disease, hospitalization, and death among persons infected following vaccination [4, 5, 13, 17]. Clinical trial endpoints utilized here were for prevention of symptomatic infection; the effectiveness of authorized vaccines at preventing asymptomatic SARS-CoV-2 infection is still unclear but preliminary reports suggest the mRNA vaccines were >90% effective at preventing infection prior to circulation of the Delta variant [13, 14, 17, 21, 25]. We did not incorporate asymptomatic infections into these calculations, nor did we update efficacy inputs to reflect decreased vaccine effectiveness against the Delta variant, which was just beginning to circulate at the end of this study period. With decreased vaccine effectiveness and increasing numbers of vaccinated persons, the expected numbers of symptomatic vaccine breakthrough cases will represent a larger proportion of total COVID-19 cases.

The analytic approach described here is based on several assumptions and limitations that affect how our results should be interpreted. First, this approach is based on reported case counts and does not account for the population at-risk, susceptibility of persons previously infected, or duration of immunity following vaccination. Second, these calculations assume that vaccinated and unvaccinated people have the same risk of exposure to SARS-CoV-2, which may not be true at the population level. Third, we define vaccine breakthrough infections as those occurring more than two weeks after completion of vaccination; these figures do not include people who may become infected following partial vaccination or prior to 14 days following completion of vaccination. Fourth, reported COVID-19 case counts stratified by patient age are not available from all states [12]. Our assumption that adults comprise 87.4% of reported cases and persons aged ≥12 years comprise 93.7% of total cases reflects cumulative trends since the beginning of the pandemic; these data may not reflect the age distribution during the weeks included here and only approximate the number of COVID-19 cases occurring among vaccine eligible people. If the proportion of total cases occurring among the vaccine eligible population decreased over time, our assumptions would yield an overestimate of vaccine breakthrough cases. Lastly, reporting delays among both COVID-19 case and vaccine administration data vary, and data are often updated retrospectively. Therefore, the figures used for these calculations should be viewed as approximations. Collectively, because of these limitations, the specific estimated counts should be interpreted with caution. Nevertheless, they provide useful context for guiding expectations.

Risk reduction provided by any vaccination is inherently relative, and the number of cases among vaccinated persons assuming equal exposure as unvaccinated persons is directly linked to vaccination coverage and disease incidence. Even with highly effective vaccines, given the large number of people being vaccinated in the United States and high levels of SARS-CoV-2 transmission in many parts of the country, hundreds of thousands of symptomatic vaccine breakthrough infections are expected, and will continue to accumulate amid high-levels of SARS-CoV-2 circulation. The methods described here can be used by public health officials to determine if the frequency of vaccine breakthrough infections reported in their jurisdictions are consistent with expectations based on vaccine efficacy from clinical trials. Furthermore, public health messaging regarding expected vaccine breakthrough infections is important to assure the public that this is expected, is not cause for alarm, and does not indicate that vaccines are not preventing severe COVID-19.

Vaccine breakthrough infections are expected to continue to accumulate amid ongoing widespread community transmission of SARS-CoV-2 and high vaccination coverage. However, the number of COVID-19 cases, hospitalizations, and deaths prevented among vaccinated persons will far exceed the numbers of vaccine breakthrough infections. CDC continues to collaborate with public health officials nationwide to monitor COVID-19 trends among vaccine persons and to identify unexpected trends in characteristics of people with vaccine breakthrough infections or patterns associated with infecting strains.

## Supporting information

S1 File Statistical methods detail. (DOCX)

S2 File Publicly available input data. (XLSX)

S3 File Framework for spreadsheet-based calculator. (XLSX)
