# Supplementary material for: Estimating the number of symptomatic SARS-CoV-2 infections among vaccinated individuals in the United States—January–July, 2021
Source: PLoS One. 2022 Mar 9;17(3):e0264179. doi: 10.1371/journal.pone.0264179 (PMC8906607; doi:10.1371/journal.pone.0264179)
Supplement: S1 File — (DOCX) [file pone.0264179.s001.docx]

**Supplemental Statistical Methods**

1. **Derivation of Expected Number of Breakthrough Cases and 95% Confidence Intervals**

We use the following notation when calculating the expected number of COVID-19 vaccine breakthrough cases and the accompanying 95% confidence interval. Let C denote the weekly COVID-19 case counts and V the vaccination group: 0 for unvaccinated (or not fully vaccinated), 1 for the single-dose COVID-19 vaccine (Janssen/Johnson & Johnson), and 2 for the two-dose mRNA vaccine (Moderna/Pfizer-BioNTech). (The mRNA vaccines were grouped together for this analysis because of their highly similar vaccine efficacy and comparable timing of authorization.) Let %V_j_ denote the percent of the population in group *j* (%V_0_, %V_1_, %V_2_ with %V_0_ + %V_1_ + %V_2_ = 1). We assume that vaccine efficacy (VE) is calculated as (1 – RR) where RR is the ratio of confirmed cases of COVID-19 per 1000 person-years in the treatment group divided by placebo group. The RRs for the two vaccine groups were calculated in two separate Poisson regression models that incorporated total cases and follow-up time for the treatment and placebo groups in each clinical trial; one model used data from the Janssen clinical trial [6] and the other used data from both the Moderna and Pfizer-BioNTech trials [4,5]. The Poisson regression models were unadjusted for confounders (all trials were randomized). The two-dose mRNA vaccination model assumed a common treatment effect for Moderna and Pfizer-BioNTech. The Janssen RR_1_ was estimated as exp($\hat{\beta}$_1_) = 0.331 ($\hat{VE}$_1_ = 0.669), the Pfizer/Moderna RR_2_ as exp($\hat{\beta}$_2_) = 0.054 ($\hat{VE}$_2_ = 0.946), and RR_0_ specified as 1 (VE_0_ = 0) for the unvaccinated.

We used Bayes Theorem to calculate the probabilities Pr (V = v_1_ | C) and Pr (V = v_2_ | C), the respective probabilities that a person who is a reported COVID-19 case on any given day was previously vaccinated with Janssen or Moderna/Pfizer-BioNTech. Thus,

Pr (V = v_1_ | C) = [ Pr(C | V = v_1_) * Pr(v_1_) ] / [ Pr(C) ] =

[ Pr(C | V = v_1_) * Pr(v_1_)] / { [ Pr(C | V = v_0_) * Pr(v_0_ )] + [ Pr(C | V = v_1_) * Pr(v_1_) ] + [ Pr(C | V = v_2_) * Pr(v_2_) ] } = [ Pr(C | V = v_1_) * %V_1_ ] / { [ Pr(C | V = v_0_) * %V_0_ ] + [ Pr(C | V = v_1_) * %V_1_ ] + [ Pr(C | V = v_2_) * %V_2_ ] }

= [ (1 – VE_1_) * %V_1_ ] / { [ (1 – VE_0_) * %V_0_ ] + [ (1 – VE_1_) * %V_1_ ] + [ (1 – VE_2_) * %V_2_ ] }

= [ (1 – VE_1_) * %V_1_ ] / { %V_0_ + [ (1 – VE_1_) * %V_1_ ] + [ (1 – VE_2_) * %V_2_ ] }

= [*RR_1_* * %V_1_ ] / { %V_0_ + [ *RR_1_* * %V_1_ ] + [ *RR_2_* * %V_2_ ] }

and following the same derivation:

Pr (V = v_2_ | C) = [ *RR_2_* * %V_2_ ] / { %V_0_ + [ *RR_1_* * %V_1_ ] + [ *RR_2_* * %V_2_ ] }.

Therefore, the estimated number of Covid-19 breakthrough cases in a day is

C { ($\hat{RR}$_1_ * %V_1_ ) + ($\hat{RR}$_2_ * %V_2_ ) } / { %V_0_ + ($\hat{RR}$_1_ * %V_1_ ) + ($\hat{RR}$_2_ * %V_2_) }

= C { (exp($\hat{\beta}$_1_) * %V_1_ ) + (exp($\hat{\beta}$_2_) * %V_2_ ) } / { %V_0_ + (exp($\hat{\beta}$_1_) * %V_1_ ) + (exp($\hat{\beta}$_2_) * %V_2_) }. Eq. (1)

We assumed that C, %V_0_ , %V_1_ , and %V_2_ are constants when obtaining the variance of the estimated number of COVID-19 vaccine breakthrough cases and corresponding 95% confidence interval. We account for the precision of $\hat{VE}$_1_ ($\hat{RR}$_1_) and of $\hat{VE}$_2_ ($\hat{RR}$_2_). From the Poisson regression models we obtained exp($\hat{\beta}$_1_) = $\hat{RR}$_1_ and exp($\hat{\beta}$_2_) = $\hat{RR}$_2_ with Var($\hat{\beta}$_1_) = 0.01149, Var($\hat{\beta}$_2_) = 0.05551, and Cov($\hat{\beta}$_1_, $\hat{\beta}$_2_) = 0 (subjects in the vaccine trials are assumed independent). By the Delta Method, the variance of the estimated number of expected COVID-19 vaccine breakthrough cases is

C^2^ { $\hat{RR}$_1_ %V_0_ %V_1_ / { [ %V_0_ + ($\hat{RR}$_1_ * %V_1_ ) + ($\hat{RR}$_2_ * %V_2_) ] ** 2 }^2^ } Var($\hat{\beta}$_1_) +

C^2^ { $\hat{RR}$_2_ %V_0_ %V_2_ / { [ %V_0_ + ($\hat{RR}$_1_ * %V_1_ ) + ($\hat{RR}$_2_ * %V_2_) ] ** 2 }^2^ } Var($\hat{\beta}$_2_).

The corresponding 95% confidence interval is calculated as the expected breakthrough cases ± (1.96 * √Variance).

1. **Simulations to assess 95% confidence interval coverage**

A simulation trial was conducted to assess the coverage of the 95% confidence intervals (CI) for the estimated number of vaccine breakthrough cases. The daily number of COVID-19 cases in the vaccine eligible population was specified as 100,000. The percentage of persons vaccinated with 1- and 2-dose regimens (%V_1_ and %V_2_ as denoted above) were varied to reflect differences in vaccine rollout. The percentage of the eligible population that was unvaccinated (%V_0_) was calculated as 1 - %V_1_  - %V_2_ . The RR for each group was specified from the vaccine trials as above: exp($\hat{\beta}$_1_) = $\hat{RR}$_1_ = 0.331 ($\hat{VE}$_1_ = 0.669); exp($\hat{\beta}$_2_) = $\hat{RR}$_2_ = 0.054 ($\hat{VE}$_2_ = 0.946) with Var($\hat{\beta}$_1_) = 0.01149 and Var($\hat{\beta}$_2_) = 0.05551. The value of RR_0_ = 1 (VE_0_ = 0) for the unvaccinated. For each scenario, let Q = 10,000 denote the number of simulated data sets.

The probability that one is a vaccine breakthrough case was calculated for each scenario and multiplied by 100,000 to calculate the expected number of vaccine breakthrough cases. Risk ratios for the 1- and 2-dose vaccine groups (RR_1_ and RR_2_ ) were independently (Cov($\hat{\beta}$_1_, $\hat{\beta}$_2_) = 0) randomly generated based on the normality assumption for $\hat{\beta}$_1_ and $\hat{\beta}$_2_ . The number of vaccine breakthrough cases for each data set was calculated based on equation 1, the binomial distribution, and the randomly generated RR_1_ and RR_2_. The corresponding 95% CIs were calculated and the coverage rate for each scenario was calculated as the number of times that the 95% CI covered the expected number of breakthrough cases divided by the 10,000 trials. The number of times that the 95% CI was above or below the expected number of vaccine breakthrough cases was also calculated and divided by 10,000. The coverage rate was uniformly close to 95%, ranging from 0.945 to 0.952 across the 15 scenarios. There was a slight tendency for the confidence intervals to fall below the expected number of vaccine breakthrough cases compared to falling above it.

| %V2 | %V1 | %V0 | Coverage Rate | High | Low |
| --- | --- | --- | --- | --- | --- |
| 0.10 | 0 | 0.90 | 0.948 | 0.027 | 0.026 |
| 0.15 | 0 | 0.85 | 0.952 | 0.028 | 0.020 |
| 0.20 | 0 | 0.80 | 0.945 | 0.026 | 0.029 |
| 0.20 | 0.05 | 0.75 | 0.947 | 0.027 | 0.026 |
| 0.25 | 0.05 | 0.70 | 0.947 | 0.026 | 0.027 |
| 0.30 | 0.05 | 0.65 | 0.947 | 0.027 | 0.027 |
| 0.35 | 0.05 | 0.60 | 0.951 | 0.022 | 0.027 |
| 0.40 | 0.10 | 0.50 | 0.953 | 0.022 | 0.025 |
| 0.45 | 0.10 | 0.45 | 0.946 | 0.022 | 0.031 |
| 0.50 | 0.10 | 0.40 | 0.947 | 0.022 | 0.031 |
| 0.55 | 0.10 | 0.35 | 0.949 | 0.022 | 0.029 |
| 0.60 | 0.10 | 0.30 | 0.949 | 0.021 | 0.030 |
| 0.60 | 0.15 | 0.25 | 0.951 | 0.017 | 0.032 |
| 0.65 | 0.15 | 0.20 | 0.949 | 0.019 | 0.032 |
